# Mortality after admission for acute myocardial infarction in Aboriginal and non-Aboriginal people in New South Wales, Australia: a multilevel data linkage study

**DOI:** 10.1186/1471-2458-12-281

**Published:** 2012-04-10

**Authors:** Deborah A Randall, Louisa R Jorm, Sanja Lujic, Aiden J O’Loughlin, Timothy R Churches, Mary M Haines, Sandra J Eades, Alastair H Leyland

**Affiliations:** 1 School of Medicine, University of Western Sydney, Narellan Road, Campbelltown, NSW, Australia; 2 The Sax Institute, Quay Street, Sydney, NSW, Australia; 3 Baker IDI Heart and Diabetes Institute, Commercial Road, Melbourne, Victoria, Australia; 4 Medical Research Council/Chief Scientist Office Social and Public Health Sciences Unit, Lilybank Gardens, Glasgow, UK

**Keywords:** Hospital performance, Acute myocardial infarction, Ischaemic heart disease, Aboriginal health, Health outcomes, Multilevel modelling, Data linkage

## Abstract

**Background:**

Heart disease is a leading cause of the gap in burden of disease between Aboriginal and non-Aboriginal Australians. Our study investigated short- and long-term mortality after admission for Aboriginal and non-Aboriginal people admitted with acute myocardial infarction (AMI) to public hospitals in New South Wales, Australia, and examined the impact of the hospital of admission on outcomes.

**Methods:**

Admission records were linked to mortality records for 60047 patients aged 25–84 years admitted with a diagnosis of AMI between July 2001 and December 2008. Multilevel logistic regression was used to estimate adjusted odds ratios (AOR) for 30- and 365-day all-cause mortality.

**Results:**

Aboriginal patients admitted with an AMI were younger than non-Aboriginal patients, and more likely to be admitted to lower volume, remote hospitals without on-site angiography. Adjusting for age, sex, year and hospital, Aboriginal patients had a similar 30-day mortality risk to non-Aboriginal patients (AOR: 1.07; 95% CI 0.83-1.37) but a higher risk of dying within 365 days (AOR: 1.34; 95% CI 1.10-1.63). The latter difference did not persist after adjustment for comorbid conditions (AOR: 1.12; 95% CI 0.91-1.38). Patients admitted to more remote hospitals, those with lower patient volume and those without on-site angiography had increased risk of short and long-term mortality regardless of Aboriginal status.

**Conclusions:**

Improving access to larger hospitals and those with specialist cardiac facilities could improve outcomes following AMI for all patients. However, major efforts to boost primary and secondary prevention of AMI are required to reduce the mortality gap between Aboriginal and non-Aboriginal people.

## Background

The health of Aboriginal and Torres Strait Islander Australians is worse than that of other Australians across every conceivable health indicator [1]. The determinants of the disproportionate ill-health among Aboriginal people include higher levels of behavioural, biomedical and psychosocial risk factors, in combination with lesser access to appropriate health services and lower socio-economic status (SES) [1-5].

While the determinants are complex, the results are clear – Aboriginal Australians have a burden of disease which is two-and-a-half times that of non-Aboriginal Australians [1], and an estimated gap in life expectancy that is greater than that in other developed countries [6]. Ischaemic heart disease (IHD) accounts for 14% of the gap in burden of disease [2], and Aboriginal Australians have higher age-adjusted rates of incidence, hospital admission and mortality for acute myocardial infarction (AMI), the acute form of IHD [3,7-9]. While several studies have compared rates of invasive interventions [7,9-11], none has quantified the impact of hospital care on variations in short-term and long-term outcomes for Aboriginal and non-Aboriginal people after admission for AMI.

This study investigated short- and long-term mortality after admission for Aboriginal and non-Aboriginal residents of New South Wales (NSW) admitted to hospital with AMI and also investigated the impact of hospital of admission on outcomes.

## Methods

### Study design

Observational cohort study using linked hospital and mortality data.

### Data sources

The NSW Admitted Patients Data Collection (APDC) includes records for all NSW public and private hospital separations (hospital admissions ending in a discharge, transfer, type-change or death). Patient demographics and multiple diagnoses and procedures are recorded for each separation and coded according to the Australian modification of the International Statistical Classification of Diseases and Related Problems (diagnoses) and the Australian Classification of Health Interventions (procedures) [12]. The NSW Register of Births, Deaths and Marriages (RBDM) captures details of all deaths registered in NSW.

### Probabilistic linkage

The APDC from 1 July 2000 to 31 December 2008 was linked with the RBDM from 1 July 2000 to 31 December 2009. Personal identifiers (including full name, date of birth, sex and address) from the datasets were linked using probabilistic methods by the Centre for Health Record Linkage [13]. The researchers were supplied with de-identified APDC and RBDM data and merged these using a project-specific unique person number.

### Setting

NSW is the most populous state in Australia with an estimated 6.8 million residents in 2006, 2.2% who identify as Aboriginal and/or Torres Strait Islander [14]. Approximately 30% of Australia’s Aboriginal peoples live in NSW, the largest percentage of all the States and Territories in Australia. In 2006, 73% of the total NSW population lived in a major city [15] compared with 42% of the NSW Aboriginal population [16]. The median age of Aboriginal people in NSW in 2006 was 20.6 years [17] while the median age for non-Aboriginal people was 38.6 years [18].

### Participants

The participants were NSW residents aged 25 to 84 who were admitted to a public hospital with a primary diagnosis of acute myocardial infarction (AMI, ICD-10-AM code ‘I21’) or ischaemic heart disease (IHD, ICD-10-AM codes ‘I20’-‘I25’) with a diagnosis of AMI in the second or third diagnosis fields, and where the admission was classified as both ‘acute care’ and ‘emergency’. Only first admissions to public hospitals were included, because the linkage for private hospitals was not of the same quality as for public hospitals. The first such admission in the period July 2001 to December 2008 was chosen as the index admission for analysis, with at least a one-year clearance period for previous admissions for AMI. The cohort thus consisted of cases whose index admission was their first-ever as well as those who had an AMI admission prior to July 2000. A sensitivity analysis excluding previously-admitted cases with clearance periods of between one and four years found no significant difference in the Aboriginal to non-Aboriginal 30-day and 365-day mortality ratios. Patients were excluded if they had missing data or appeared to be duplicate admissions (244 non-Aboriginal and 3 Aboriginal records). The excluded records had the same percentage of deaths within 30 days as the final data set (9%). The final data set included 60047 patients (1183 Aboriginal, 58864 non-Aboriginal) admitted to 174 public hospitals in NSW.

### Analysis variables

The main outcomes were 30-day and 365-day all-cause mortality after hospital admission. The main variable of interest was whether the patient identified as Aboriginal. This was determined based on the standard question, “Are you of Aboriginal or Torres Strait Islander origin?”, recorded in the hospital data. In 2007, an audit was conducted and the percentage of Aboriginal and Torres Strait Islander patients correctly identified in NSW public hospitals was estimated to be 88% [19]. While identification is thought to have improved over time, there were no audits previously published for NSW [20]. However, the Australian Institute of Health and Welfare used an under-identification factor of 30% to correct expenditure data for 1998–99 and 2001–02 for NSW hospital data [20]. Probabilistic linkage provided opportunities for identification across the entire admission history for each individual but in the absence of an external source of Aboriginal status to validate identification algorithms, we defined Aboriginal people in our study (Aboriginal and/or Torres Strait Islander) based on the most recent public hospital admission recorded for each person. This was thought to be the most accurate method due to improvements in identification over time [19,20]. A sensitivity analysis was carried out using two alternative definitions: identified as Aboriginal on every admission (‘all admissions’) or on at least one admission (‘ever identified’).

Comorbidities were measured with the Ontario AMI mortality prediction rule (OAMIMPR) [21] conditions, developed in Ontario, Canada for risk adjustment specifically after AMI admission, and were supplemented with additional Charlson Comorbidity Index conditions [22] that had a significant age-, sex- and year-adjusted association with 30-day or 365-day mortality. All comorbidities were collected with a one-year look-back that included any comorbid conditions recorded on the APDC for each person for a full year before the AMI admission as well as on the admission record. Socio-economic status was classified using the ABS Socio-Economic Index for Areas Index of Relative Social Disadvantage (SEIFA IRSD) based on Statistical Local Area (SLA) of residence, and divided into population quintile groups. Remoteness of residence was ascertained using the Accessibility/Remoteness Index of Australia (ARIA+) for SLA of residence, grouped into four categories (major city, inner regional, outer regional and remote/very remote). The hospital of analysis was the first hospital of admission in the AMI admission episode. There were three hospital-level variables: hospital remoteness (ARIA + group of the hospital based on postcode), hospital size (the average number of all acute admissions per year between 2001 and 2008, calculated for each hospital and divided into five groups at the 50th, 75th, 85th and 95th percentiles for hospitals), and the presence or absence of on-site cardiac angiography facilities.

### Statistical analysis

Characteristics of Aboriginal and non-Aboriginal people admitted with AMI were compared using χ^2^ tests. Comorbidities were additionally compared using age-, sex- and year-adjusted prevalence ratios calculated using a log-Poisson model. A series of multilevel logistic regression models with 60047 AMI patients clustered within 174 hospitals investigated: the relative odds of 30-day and 365-day mortality after admission for Aboriginal people compared with non-Aboriginal people with stepwise adjustment of individual and hospital factors; how much of the variation in mortality related to the hospital of admission; what individual characteristics are associated with 30-day and 365-day mortality; and what hospital characteristics might explain residual variation between hospitals. The number of AMI patients per hospital in the final models ranged from 1 to 2691, with a median of 65. Only 5% of hospitals had two or fewer patients. Multilevel modelling accounts for the clustering of patients within hospitals and also partitions the residual variation into the between-hospital variation and within-hospital variation [23]. All multilevel models had a random intercept allowing the hospital mortality rate to vary, and we also tested random slope models to see if the odds ratio for Aboriginal status varied between hospitals. The hospital-level variance can be expressed as a percentage of the total variance, also called the intraclass correlation coefficient (ICC), or can be converted into a median odds ratio (MOR), which is the median of the odds ratios of pair-wise comparisons of patients with identical characteristics taken from randomly chosen hospitals [24]. Data analyses were carried out using SAS 9.1.3 [25] and MLwiN 2.22 [26].

### Ethics approval

Approval for the study was given by the NSW Population and Health Services Research Ethics Committee, the Aboriginal Health and Medical Research Council of NSW Ethics Committee, and the University of Western Sydney Human Research Ethics Committee.

## Results

### Patient characteristics

**Figure 1:**
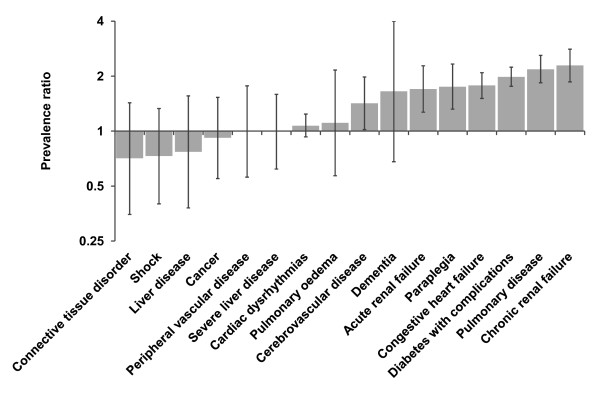
Prevalence ratio adjusted for age, sex and year of admission, for Aboriginal compared with non-Aboriginal people with AMI. A value over 1 indicates that Aboriginal people have a higher prevalence of the condition, and a value under 1 indicates that non-Aboriginal people have a higher prevalence. Comorbid conditions calculated with one-year look-back, including comorbidities on current admission and any admissions in the previous year.

Aboriginal patients with AMI were significantly younger than non-Aboriginal patients with just over half of the Aboriginal patients aged 25–54 years compared with only one-fifth of non-Aboriginal patients (Table 1). Aboriginal patients were also more likely to be female, more likely to be living in an area classified as most disadvantaged, and more likely to be living in an outer regional or remote area. Aboriginal patients were significantly less likely to be admitted to a major city hospital, a hospital with 18400 or more average acute admissions per year, or one with on-site angiography facilities. Due to the marked demographic differences, age-, sex- and year-adjusted prevalence ratios were calculated to compare the prevalence of comorbidities. These showed that Aboriginal patients were more likely than non-Aboriginal patients of the same age, sex and year of admission to have acute and chronic renal failure, paraplegia, congestive heart failure, diabetes with complications, and pulmonary disease (Figure 1).

**Table 1:** Individual and hospital characteristics of Aboriginal and non-Aboriginal people admitted with acute myocardial infarction

	**Aboriginal (n = 1183)**		**Non- Aboriginal (n = 58877)**		**χ**^**2**^**p-value**
	***N***	**%**	***N***	**%**	
**Individual characteristic**					
Age group					
25-34	47	4.0	421	0.7	<.001
35-44	257	21.7	2829	4.8	
45-54	360	30.4	8579	14.6	
55-64	265	22.4	13144	22.3	
65-74	180	15.2	15410	26.2	
75-84	74	6.3	18481	31.4	
Sex					
Male	727	61.5	39950	67.9	<.001
Female	456	38.5	18914	32.1	
Comorbid conditions^a^					
Diabetes with complications	279	23.6	8903	15.1	<.001
Cardiac dysrhythmias	185	15.6	12539	21.3	<.001
Congestive heart failure	154	13.0	8350	14.2	.254
Pulmonary disease	136	11.5	5236	8.9	.002
Chronic renal failure	96	8.1	4143	7.0	.152
Acute renal failure	47	4.0	2729	4.6	.282
Cerebrovascular disease	36	3.0	2552	4.3	.030
Paraplegia	25	2.1	1341	2.3	.707
Cancer	15	1.3	1773	3.0	0.01
Peripheral vascular disease	12	1.0	1108	1.9	.029
Shock	11	0.9	1195	2.0	.008
Pulmonary oedema	9	0.8	685	1.2	.199
Connective tissue disorder	8	0.7	743	1.3	.073
Dementia	5	0.4	506	0.9	.105
Severe liver disease	2	0.2	103	0.2	.962
Liver disease	1	0.1	113	0.2	.615
Socio-economic status^b^					
1^st^ quintile - least disadvantaged	23	1.9	7832	13.3	<.001
2^nd^ quintile	109	9.2	9946	16.9	
3^rd^ quintile	186	15.7	12726	21.6	
4^th^ quintile	299	25.3	13102	22.3	
5^th^ quintile - most disadvantaged	566	47.8	15258	25.9	
Remoteness of residence^c^					
Major city	307	26.0	34695	58.9	<.001
Inner regional	359	30.3	16319	27.7	
Outer regional	366	30.9	7300	12.4	
Remote/very remote	151	12.8	550	0.9	
**Hospital characteristic**					
Remoteness of hospital^c^					
Major city	389	32.9	39456	67.0	<.001
Inner regional	247	20.9	11384	19.3	
Outer regional	414	35.0	7297	12.4	
Remote/very remote	133	11.2	726	1.2	
Average acute admissions per year					
Less than 1200	88	7.4	1245	2.1	<.001
1200-3899	182	15.4	3730	6.3	
3900-7084	138	11.7	3842	6.5	
7085-18399	443	37.4	19977	33.9	
18400 or more	332	28.1	30070	51.1	
On-site angiography					
Yes	315	26.6	25694	43.6	<.001
No	868	73.4	33170	56.4	

^a^ Comorbid conditions with one-year look-back, including comorbidities on current admission and any admissions in the previous year.

^b^ Socio-Economic Indices for Areas (SEIFA) Index of Relative Socio-Economic Disadvantage population quintiles based on statistical local area of residence.

^c^ Accessibility/Remoteness Index of Australia (ARIA+) based on statistical local area of residence for individuals or hospital postcode for hospitals.

### Short- and long-term mortality after admission

Of the 1183 Aboriginal patients admitted with AMI, 70 died within 30 days of admission (5.9%) and 127 died within one year of admission (10.7%). Of the 58864 non-Aboriginal patients admitted with AMI, 5474 died within 30 days (9.3%) and 9148 died within one year (15.2%). When accounting only for hospital of admission through the random intercept multilevel model, Aboriginal patients with AMI had lower odds of dying within 30 days than non-Aboriginal patients (odds ratio (OR) 0.61; 95% CI 0.48-0.78; Table 2, Model 1A). However, after adjusting for age, sex and year of admission there was no significant difference in 30-day mortality between Aboriginal and non-Aboriginal AMI patients (AOR 1.07; 95% CI 0.83-1.37; Model 2A). Accounting for comorbidities, remoteness of residence, and socio-economic status (Model 5A) reduced the adjusted odds ratio to 0.95 (0.73-1.23), indicating no significant difference in 30-day mortality. A random slope effect for Aboriginal status was tested, but there was no significant variation in the Aboriginal to non-Aboriginal 30-day mortality ratio across hospitals.

**Table 2:** Relative odds of 30-day and 365-day mortality for Aboriginal compared with non-Aboriginal people with stepwise adjustment for covariates

**30-day mortality models**				
**Model**	**Adjusted for:**	**OR**	**95% CI**	**p-value**
1A	Hospital of admission^a^	0.61	0.48-0.78	<.001
	**Individual covariates**			
2A	+ Age group, sex, year of admission	1.07	0.83-1.37	.612
3A	+ Comorbid conditions^b^	0.98	0.76-1.27	.886
4A	+ Remoteness of residence^c^	0.95	0.73-1.24	.728
5A	+ Socio-economic status^d^	0.95	0.73-1.23	.684
	**Hospital covariates**^**e**^			
6A	+ Remoteness of hospital^c^	0.94	0.72-1.22	.617
7A	+ Average acute admissions per year (- Remoteness of hospital)	0.95	0.73-1.23	.676
8A	+ On-site angiography (- Average acute admissions per year)	0.94	0.73-1.23	.665
**365-day mortality models**				
**Model**	**Adjusted for:**	**OR**	**95% CI**	**p-value**
1B	Hospital of admission^a^	0.64	0.53-0.77	<.001
	**Individual covariates**			
2B	+ Age group, sex, year of admission	1.34	1.10-1.63	.003
3B	+ Comorbid conditions^b^	1.12	0.91-1.38	.282
4B	+ Remoteness of residence^c^	1.11	0.90-1.37	.317
5B	+ Socio-economic status^d^	1.11	0.90-1.36	.345
	**Hospital covariates**^**e**^			
6B	+ Remoteness of hospital^c^	1.09	0.89-1.35	.401
7B	+ Average acute admissions per year (- Remoteness of hospital)	1.11	0.90-1.36	.336
8B	+ On-site angiography (- Average acute admissions per year)	1.10	0.90-1.36	.350

OR, odds ratio; CI, confidence interval.

^a^ Hospital of admission adjusted for in a two-level random intercept model with patients nested within hospitals.

^b^ Comorbid conditions with one-year look-back, including comorbidities on current admission and any admissions in the previous year.

^c^ Accessibility/Remoteness Index of Australia (ARIA+) based on statistical local area of residence for individuals or hospital postcode for hospitals.

^d^ Socio-Economic Indices for Areas (SEIFA) Index of Relative Socio-Economic Disadvantage population quintiles based on statistical local area of residence.

^e^ Hospital covariates added one at a time to the adjusted models.

The unadjusted results for 365-day mortality were similar to the 30-day model: Aboriginal patients were less likely to die within 365 days of admission than non-Aboriginal patients admitted to the same hospital (OR 0.64; 95% CI 0.53-0.77; Table 2, Model 1B). However, after adjusting for age, sex, and year of admission, Aboriginal patients had significantly higher odds of dying within 365 days than non-Aboriginal patients admitted to the same hospital (AOR 1.34, 95% CI 1.10-1.63; Model 2B). Again, there was no random slope effect for Aboriginal status in this model. After comorbidities were accounted for there was no longer a significant difference between Aboriginal and non-Aboriginal 365-day mortality (AOR 1.12, 95% CI 0.91-1.38; Model 3B).

In the fully-adjusted individual-level model for 30-day mortality (Model 5A), the percentage of unexplained variation due to the hospital of admission (or the Intraclass correlation coefficient) was 2.72%. This can be expressed as a median odds ratio (MOR) of 1.34. In the fully-adjusted 365-day mortality model (Model 5B), the hospital of admission accounted for 2.58% of the unexplained variation in the outcome (MOR 1.33).

Table 3 shows odds ratios for selected individual covariates from the fully-adjusted individual-level models (Models 5A and 5B). There were no significant differences in 30-day or 365-day mortality between males and females. Older age was strongly related to both 30-day and 365-day mortality. Area of residence was not a significant predictor of 30-day or 365-day mortality, but remoteness was already being largely accounted for by adjusting for hospital of admission. Living in an area classified as the most disadvantaged was associated with higher 30-day mortality, and there was higher 365-day mortality in all the more disadvantaged quintiles compared with the least disadvantaged group. Of the comorbidities included in the model, shock was most strongly related to risk of morality, particularly 30-day mortality. Severe liver disease, cardiac dysrhythmias, dementia, cancer and acute renal failure were associated with at least a doubling in the odds of dying within 30 and 365 days of admission. Most of the other comorbidities were significantly associated with an increased risk of either 30-day or 365-day mortality. Diabetes with complications was related to a slightly lower risk of 30-day mortality but a slightly higher risk of 365-day mortality.

**Table 3:** Adjusted odds ratios for selected individual covariates for 30-day and 365-day mortality multilevel models

	**30-day mortality**			**365-day mortality**		
	**AOR**	**95% CI**	**p-value**	**AOR**	**95% CI**	**p-value**
Sex						
Male (ref)	1.00		.211	1.00		.853
Female	1.04	0.98-1.11		1.01	0.95-1.06	
Age group						
25-34	1.20	0.79-1.83	<.001	0.92	0.61-1.37	<.001
35-44	0.73	0.59-0.91		0.58	0.48-0.72	
45-54	0.80	0.70-0.92		0.74	0.66-0.83	
55-64 (ref)	1.00			1.00		
65-74	1.52	1.38-1.68		1.70	1.57-1.85	
75-84	2.30	2.10-2.53		3.09	2.86-3.34	
Comorbid conditions^a^						
Shock	11.54	10.12-13.16	<.001	7.94	6.94-9.09	<.001
Severe liver disease	3.80	2.36-6.11	<.001	2.60	1.63-4.17	<.001
Cardiac dysrhythmias	2.69	2.53-2.86	<.001	2.18	2.06-2.30	<.001
Dementia	2.30	1.87-2.84	<.001	2.72	2.25-3.30	<.001
Cancer	2.18	1.92-2.47	<.001	4.45	4.00-4.95	<.001
Acute renal failure	2.02	1.82-2.25	<.001	2.00	1.82-2.19	<.001
Cerebrovascular disease	1.53	1.33-1.77	<.001	1.47	1.29-1.66	<.001
Peripheral vascular disease	1.32	1.11-1.57	<.001	1.30	1.12-1.51	<.001
Pulmonary disease	1.25	1.14-1.37	<.001	1.56	1.45-1.67	<.001
Pulmonary oedema	1.22	0.99-1.51	.056	1.50	1.26-1.80	<.001
Congestive heart failure	1.20	1.12-1.30	<.001	1.81	1.70-1.92	<.001
Paraplegia	1.08	0.89-1.31	.424	1.43	1.21-1.69	<.001
Chronic renal failure	1.06	0.95-1.17	.282	1.56	1.44-1.70	<.001
Liver disease	0.97	0.55-1.69	.913	2.42	1.55-3.79	<.001
Diabetes with complications	0.89	0.82-0.97	.007	1.10	1.03-1.18	.003
Connective tissue disorder	0.81	0.62-1.04	.096	1.18	0.97-1.43	.090
Remoteness of residence^b^						
Major city (ref)	1.00		.223	1.00		.685
Inner regional	0.95	0.86-1.06		0.95	0.87-1.04	
Outer regional	1.08	0.94-1.24		1.00	0.89-1.12	
Remote/very remote	1.16	0.84-1.58		0.96	0.73-1.27	
Socio-economic status^c^						
1^st^ quintile - least disadvantaged (ref)	1.00		.033	1.00		<.001
2^nd^ quintile	1.15	0.99-1.32		1.11	0.98-1.25	
3^rd^ quintile	1.10	0.95-1.28		1.18	1.04-1.34	
4^th^ quintile	1.17	1.00-1.36		1.23	1.08-1.39	
5^th^ quintile - most disadvantaged	1.27	1.08-1.48		1.32	1.16-1.51	

Adjusted odds ratios for selected covariates from Model 5A for 30-day mortality and Model 5B for 365-day mortality.

AOR, adjusted odds ratio; CI, confidence interval; Ref, referent group in the analysis.

^a^ Comorbid conditions with one-year look-back, including comorbidities on current admission and any admissions in the previous year.

^b^ Accessibility/Remoteness Index of Australia (ARIA+) based on statistical local area of residence.

^c^ Socio-Economic Indices for Areas (SEIFA) Index of Relative Socio-Economic Disadvantage population quintiles based on statistical local area of residence.

Table 4 shows the relative odds of 30-day and 365-day mortality for the hospital characteristics, added one at a time to the fully adjusted individual-level model. Hospital remoteness was a significant predictor of both 30-day and 365-day mortality; those patients admitted to an outer regional or a remote hospital had significantly higher odds of mortality than those admitted to a major city hospital. Those admitted to hospitals with 7084 or less acute patients per year had higher odds of both 30-day and 365-day mortality than those admitted to hospitals with higher numbers of acute admissions per year and there was a significant trend across groups (P < 0.001 for both 30-day and 365-day models). Those admitted to a hospital with on-site angiography had lower odds of 30-day and 365-day mortality than those admitted to a hospital without these facilities. When all three hospital level variables were added they accounted for 37% of the hospital level variation in both the 30-day and 365-day mortality models.

**Table 4:** Adjusted odds ratios for selected hospital covariates for 30-day and 365-day mortality multilevel models

	**30-day mortality**			**365-day mortality**		
	**AOR**	**95% CI**	**p-value**	**AOR**	**95% CI**	**p-value**
**Added into adjusted**^**a**^**model separately**						
Remoteness of hospital^b^						
Major city (ref)	1.00		<.001	1.00		<.001
Inner regional	1.15	0.94-1.41		1.16	0.97-1.39	
Outer regional	1.56	1.26-1.94		1.54	1.27-1.87	
Remote/very remote	1.83	1.19-2.81		1.79	1.22-2.61	
Average acute admissions per year						
Less than 1200	2.03	1.57-2.62	<.001	1.98	1.58-2.49	<.001
1200-3899	1.72	1.39-2.13		1.55	1.28-1.88	
3900-7084	1.36	1.08-1.70		1.32	1.07-1.62	
7085-18399	1.14	0.96-1.35		1.14	0.97-1.34	
18400 or more (ref)	1.00			1.00		
On-site angiography						
Yes	0.74	0.64-0.86	<.001	0.72	0.63-0.83	<.001
No (ref)	1.00			1.00		

AOR, adjusted odds ratios; CI, confidence interval. Ref, referent group in the analysis.

^a^ Adjusted for Aboriginal status, age, sex, year of admission, comorbidities, remoteness of residence, socio-economic status, and a random hospital intercept, with hospital covariates added in one at a time to the model.

^b^ Accessibility/Remoteness Index of Australia (ARIA+) based on hospital postcode.

### Sensitivity analysis

The two alternative classifications for Aboriginal status, ‘ever identified’ and ‘all admissions’, identified 1479 (2.5%) and 631 (1.1%) AMI patients as Aboriginal, respectively, compared with the ‘most recent’ which identified 1183 (2.0%) of patients as Aboriginal. When entered into the fully-adjusted individual-level models, the ‘ever identified’ definition produced similar results to the ‘most recent’ definition, but the ‘all admissions’ definition resulted in higher odds of both 30-day and 365-day mortality for Aboriginal compared with non-Aboriginal patients (Table 5)..

**Table 5:** Relative odds of 30-day and 365-day mortality by different algorithms for identifying Aboriginal people in the hospital data

	**30-day mortality**			**365-day mortality**		
	**AOR**	**(95% CI)**	**p-value**	**AOR**	**(95% CI)**	**p-value**
Most recent^a^						
Non-Aboriginal (ref)	1.00		.684	1.00		.345
Aboriginal	0.95	0.73-1.23		1.11	0.90-1.36	
Ever identified^b^						
Non-Aboriginal (ref)	1.00		.582	1.00		.510
Aboriginal	0.94	0.74-1.18		1.06	0.88-1.28	
All admissions^c^						
Non-Aboriginal (ref)	1.00		.005	1.00		<.001
Aboriginal	1.55	1.14-2.10		1.61	1.25-2.07	

AOR, adjusted odds ratios, adjusting for age, sex, year of admission, comorbidities, remoteness of residence, socio-economic status.

CI, confidence interval. Ref, referent group in the analysis.

^a^ Identified as Aboriginal in their most recent public hospital admission.

^b^ Identified as Aboriginal in at least one public hospital admission.

^c^ Identified as Aboriginal on all public hospital admissions

## Discussion

Our study is, to the best of our knowledge, the first to investigate disparities in mortality outcomes between Aboriginal and non-Aboriginal people after admission for AMI in NSW, home to 30% of Australia’s Aboriginal population [14]. The overall population size and the large number of Aboriginal people residing in NSW made it possible to use multilevel modelling to examine mortality outcomes, and it is the first study of AMI hospital outcomes nationally to account for clustering of patients within hospitals and to quantify the contribution of the admitting hospital to variation in mortality outcomes.

Aboriginal and non-Aboriginal people with AMI admitted to NSW hospitals were very different. Aboriginal patients were younger, more likely to live outside of major centres and in disadvantaged areas, and more likely to be admitted to lower volume hospitals outside major centres and those without on-site angiography facilities. After adjusting for age, sex and year, they were more likely to present with comorbid conditions, including acute and chronic renal failure, diabetes, congestive heart failure and pulmonary disease. Aboriginal people in Australia have a younger age distribution than non-Aboriginal people, so it is not unexpected that Aboriginal people admitted with AMI would be younger; however, higher age-specific incidence of AMI particularly among younger Aboriginal people was recently reported by a study in Western Australia (WA) [8]. These findings and ours point to the importance of targeting the early onset of AMI among Aboriginal people and preventing or managing chronic diseases that may complicate treatment or lead to poorer long-term outcomes.

Our study found that once admitted to hospital, Aboriginal patients with AMI were less likely to die within 30 days than non-Aboriginal patients admitted to the same hospital (Table 2, Model 1A). However, this finding was explained by substantial age differences: after adjusting for age, sex and year of admission, the differences in 30-day mortality was no longer significant (Model 2A). In contrast, after adjusting for age, sex and year, Aboriginal patients had 34% higher odds of dying within one year compared with non-Aboriginal patients admitted to the same hospital (Model 2B). However, this difference was no longer significant after adjusting for selected comorbidities (Model 3B), suggesting that part of the higher one-year mortality is due to the higher comorbidity burden among Aboriginal people admitted with AMI.

Our findings regarding short-term mortality differed from those of the WA study, which reported higher post-admission 28-day mortality ratios for Aboriginal compared with non-Aboriginal patients, ranging from 1.7 in 55–74 year-old males and females to 3.6 in 25–54 year old males [8]. This discrepancy might relate to the different profile of the WA Aboriginal population (41% resident in remote or very remote areas, compared with 5% in NSW) [18], and differences in study methodology (the WA study did not account for hospital of admission).

For longer-term mortality, our findings were similar to those of a Queensland study that reported an age-adjusted risk ratio of 1.8 (95% CI, 1.5-2.2) for 365-day mortality in Aboriginal patients with AMI after admission to Queensland public hospitals [10]. We found that the significantly higher one-year mortality for Aboriginal patients did not persist after adjusting for comorbidities, but a recent study in WA found significantly higher rates of two-year cardiovascular death or recurrent AMI for Aboriginal compared with non-Aboriginal males and females after adjusting for demographic characteristics and comorbidities [27]. These findings may suggest that the Aboriginal to non-Aboriginal disparity in mortality is greater in WA than in NSW. However, it is difficult to compare these findings directly because our study had a shorter length of follow-up for all-cause mortality, adjusted for hospital of admission, and did not examine mortality and recurrent AMI as a combined outcome. An increase in the Aboriginal to non-Aboriginal mortality ratio with increasing time after discharge has been shown in the Northern Territory for those admitted with acute coronary syndrome and surviving to discharge, with the disparities in mortality appearing at six months and Aboriginal patients being about three times more likely to die than non-Aboriginal patients after four years [28]. However, caution must be taken when comparing Aboriginal peoples across Australia due to the differences in culture, geographic distribution, and access to and provision of services.

Our study showed that differences between hospitals impacted on mortality outcomes for both Aboriginal and non-Aboriginal patients. After adjustment for patient factors, 2.72% of the remaining variation in 30-day mortality was attributable to differences between hospitals. This equates to a median odds ratio of 1.34, indicating a median difference of 34% in the odds of dying between randomly chosen pairs of hospitals. Almost 40% of this hospital-level contribution to variation in mortality was explained by hospital remoteness, hospital size and cardiac facilities. Patients admitted to smaller hospitals, and those in outer regional and remote areas, had a higher risk of short-term mortality, while patients admitted to a hospital with on-site angiography facilities had a reduced risk of dying. Recently, in the United States, condition-specific hospital volume was shown to be related to 30-day post-admission mortality after AMI, up to a threshold value, which was lower for hospitals with cardiac revascularisation services (432 vs 586 AMI admissions/year) [29]. A Canadian study also found that admission to hospitals with on-site revascularisation facilities was related to improved long-term outcomes after AMI [30]. However, our findings regarding the specific impact of hospital size, remoteness and on-site angiography facilities on outcomes should be interpreted with caution, as these variables may be correlated with other unmeasured aspects of hospital quality of care. We found no variation in the Aboriginal to non-Aboriginal mortality ratio (both short- and long-term) across hospitals.

There were limitations to our study due to using administrative data not collected for research purposes. Firstly, there was limited clinical information in the hospital data for risk adjustment; however, we used the conditions adjusted for in the Ontario AMI Mortality Risk Prediction Rule developed in Canada for use with AMI and administrative hospital data [21] and supplemented this with additional conditions from the Charlson Comorbidity Index [22]. Secondly, we were not able to remove all prevalent cases from our study because there were only a total of eight and a half years of linked data available. We did, however, test various clearance periods of up to four years and found that the Aboriginal to non-Aboriginal age-and sex-adjusted mortality ratios did not appear sensitive to the length of the clearance period. Thirdly, our sensitivity analysis using different algorithms for identifying Aboriginal people highlighted the potential for apparent disparities to be influenced by how Aboriginal status is defined. The strict definition requiring patients to be identified as Aboriginal at every hospital admission identified only 1% of admissions as Aboriginal which is half as many as the ‘most recent’ algorithm but generated higher relative odds of Aboriginal mortality. This may be because those people consistently identified as Aboriginal in the APDC have poorer health than Aboriginal people not consistently identified, but it may also be because the definition included a greater proportion with only a single admission, possibly skewing the sample towards people who died post-AMI. Lastly, we did not include deaths from AMI that occurred before the patient was admitted to hospital, either sudden death or death in ambulance or Emergency Department. It is possible that Aboriginal people would be overrepresented in these early deaths from AMI, due to higher comorbidity rates or living a greater distance from the nearest hospital, but this was outside the scope of our study examining outcomes after hospital admission.

Our study and others point to the importance of prevention and early intervention to target the early onset of AMI among Aboriginal Australians. These efforts must target risk factor prevalence among Aboriginal people, including higher rates of smoking and overweight and obesity, and the earlier onset of comorbidities like diabetes and renal failure [1]. However, poor health behaviours may be a way of coping for people living under chronically stressful conditions, so psychosocial and emotional factors must also be taken into account [31,32]. Importantly, our study has demonstrated that there are gains to be made—both for Aboriginal and non-Aboriginal people—by improving access to larger hospitals and hospitals with on-site angiography or by improving the cardiac care facilities at smaller hospitals.

The population density and geographic distances in Australia pose difficult policy questions about whether it is best to transfer patients as quickly as possible to major city hospitals or whether it is efficient to increase services in less densely population areas. Our results showed that the difference in outcomes for inner regional compared with major city hospitals was small and not significant, so boosting resources in regional centres may reduce the difference altogether, and reduce travel times to cardiac facilities for those living in regional and remote areas. One challenge is to ensure that any interventions are culturally appropriate for Aboriginal patients. While transfers can be very stressful for Aboriginal people living in remote areas, an action research study concluded that small interventions such as having dedicated liaison officers in the health system could improve cultural awareness of practitioners as well as communication and continuity of care and improve outcomes for Aboriginal patients [33].

The higher mortality among Aboriginal patients in the first year after admission also highlights the importance of improved post-AMI care including appropriate medication and lifestyle interventions. This period after discharge warrants further investigation to disentangle the impacts on mortality of comorbidity burden and differences in access to, or adherence with, follow-up care and secondary prevention.

## Conclusions

Improving access to larger hospitals or those with specialist treatment facilities could improve outcomes following AMI for residents of rural and regional areas, both Aboriginal and non-Aboriginal. However, major efforts to boost primary and secondary prevention of AMI are required to reduce the mortality gap between Aboriginal and non-Aboriginal people.

## Competing interests

The authors declare that they have no competing interest.

## Authors’ contributions

DR had overall responsibility for the design of this study, data management, statistical analysis and drafting this paper. LJ initiated the IHOPE project and provided overall oversight. LJ, SL, TC, MH, SE and AL contributed to the conception and design of the IHOPE project. SL provided advice on data management and statistical analysis. AO provided advice on clinical aspects and on hospital levels of service. AL provided oversight and advice for the design and interpretation of the statistical analyses. All authors contributed to the interpretation of findings, the writing of the paper and approved the final draft.

## Pre-publication history

The pre-publication history for this paper can be accessed here:

http://www.biomedcentral.com/1471-2458/12/281/prepub
